# Outbreak of OXA-232-producing carbapenem-resistant *Klebsiella pneumoniae* ST15 in a Chinese teaching hospital: a molecular epidemiological study

**DOI:** 10.3389/fcimb.2023.1229284

**Published:** 2023-08-21

**Authors:** Xiaoyan Wu, Xiangchen Li, Junjie Yu, Mengli Shen, Chenliang Fan, Yewei Lu, Junshun Gao, Xiaosi Li, Hongsheng Li

**Affiliations:** ^1^ Department of Laboratory Medicine, the Second Affiliated Hospital of Jiaxing University, Jiaxing, Zhejiang, China; ^2^ Key Laboratory of Precision Medicine in Diagnosis and Monitoring Research, Hangzhou, Zhejiang, China

**Keywords:** carbapenem-resistant *Klebsiella pneumonia*, nosocomial epidemics, clonal transmission, whole genome sequencing, outbreak

## Abstract

**Background and Aims:**

The incidence of OXA-232-producing carbapenem-resistant *Klebsiella pneumoniae* (CRKP) has been on the rise in China over the past five years, potentially leading to nosocomial epidemics. This study investigates the first outbreak of CRKP in the Second Affiliated Hospital of Jiaxing University.

**Methods:**

Between February 2021 and March 2022, 21 clinical isolates of OXA-232-producing CRKP were recovered from 16 patients in the Second Affiliated Hospital of Jiaxing University. We conducted antimicrobial susceptibility tests, whole genome sequencing, and bioinformatics to determine the drug resistance profile of these clinical isolates.

**Results:**

Whole-genome sequencing revealed that all 21 OXA-232-producing CRKP strains belonged to the sequence type 15 (ST15) and shared similar resistance, virulence genes, and plasmid types, suggesting clonal transmission between the environment and patients. Integrated genomic and epidemiological analysis traced the outbreak to two clonal transmission clusters, cluster 1 and cluster 2, including 14 and 2 patients. It was speculated that the CRKP transmission mainly occurred in the ICU, followed by brain surgery, neurosurgery, and rehabilitation department. Phylogenetic analysis indicated that the earliest outbreak might have started at least a year before the admission of the index patient, and these strains were closely related to those previously isolated from two major adjacent cities, Shanghai and Hangzhou. Comparative genomics showed that the IncFII-type and IncHI1B-type plasmids of cluster 2 had homologous recombination at the insertion sequence sites compared with the same type of plasmids in cluster 1, resulting in the insertion of 4 new drug resistance genes, including *TEM-1*, *APH(6)-Id*, *APH(3’’)-Ib* and *sul2*.

**Conclusions:**

Our study observed the clonal spread of ST15 OXA-232-producing between patients and the hospital environment. The integration of genomic and epidemiological data offers valuable insights and facilitate the control of nosocomial transmission.

## 1 Introduction

*Klebsiella pneumoniae* is one of the leading causes of hospital-acquired infections globally (Pitout et al., 2015; David et al., 2019). Carbapenems are widely used to treat serious infections caused by multidrug-resistant (MDR) *Enterobacteriaceae*, such as *AmpC β*-lactamases and extended-spectrum *β*-lactamases (ESBLs). However, in recent years, the widespread use of carbapenems has accelerated the growth of resistant strains (Shrivastava et al., 2018). The China Antimicrobial Surveillance Network (CHINET) showed that CRKP in children increased from 2.2% in 2005 to 25.4% in 2017 and then conducted a gradually decreasing trend year by year until 2020 (Yang et al., 2023). CHINET data suggested that the CRKP isolation rate in 2021 was 42.2% in Zhejiang Province, which was the highest among regions in eastern China (Yang et al., 2023).

The mechanism of carbapenem resistance in *K. pneumoniae* mainly relies on the production of carbapenemases, which can be characterized into different enzyme types, such as KPC, NDM, VIM, IMP, and OXA-48 (Karaiskos et al., 2022). OXA-48 belonged to the class D carbapenemase, which was identified in a clinical *K. pneumoniae* isolate in 2004 (Poirel et al., 2012). OXA-232, an OXA-48-like variant, was first identified in France from a *K. pneumoniae* isolate in 2013 (Potron et al., 2013). Unlike other OXA-48-like variants, OXA-232 has weaker carbapenemase activity (Bakthavatchalam et al., 2016). However, the OXA-232 variant has the potential to cause high-level carbapenem resistance once combined with extended-spectrum *β*-lactamases or outer membrane protein mutations. Since the initial isolation of OXA-232-producing CRKP in five neonatal patients in China in 2017 (Yin et al., 2017), there have been a number of small-scale nosocomial outbreaks of the OXA-232-producing CRKP among children and elderly patients (Li et al., 2019; Han et al., 2022; Zhang et al., 2023).

The rapid evolution of *K. pneumoniae* challenges the standard infection prevention and control efforts (Chang et al., 2021). With the development of whole-genome sequencing (WGS) technology, the use of core genome multi-locus sequence typing (cgMLST) or single nucleotide polymorphism (cgSNP) in subtyping and outbreaks monitoring of bacteria has become increasingly widespread (Wyres et al., 2020). Recent studies have shown that both approaches are suitable for transmission analyses of several pathogenic bacteria, including *Staphylococcus aureus*, *Staphylococcus epidermidis*, *Streptococcus mutans*, and *K. pneumoniae* (Li et al., 2018; Humphreys and Coleman, 2019; Liu et al., 2020; Zhang et al., 2022). In addition, WGS has also been used to examine the drug-resistance mechanisms and the potential transfer of antibiotic resistance genes by plasmids (Wyres and Holt, 2018; Zong et al., 2021).

Based on the integrated analysis of genomic and epidemiological data, our study investigated the first outbreak of OXA-232-producing ST15 CRKP in a teaching hospital in China between February 2021 and March 2022. We examined the phenotypic, genotypic, phylogenetic, and epidemiological characteristics of OXA232-producing CRKP isolates. The findings of this study could provide theoretical insights for the prevention and control of nosocomial infections caused by high-risk OXA-232-producing CRKP clones.

## 2 Materials and methods

### 2.1 Bacterial isolates and clinical data collection

This study was conducted at the Second Affiliated Hospital of Jiaxing University in Jiaxing, a first-class, grade three hospital in Zhejiang province. We collected 21 clinical *K. pneumoniae* isolates from 16 inpatients in this hospital between February 2021 and March 2022. These strains were isolated from diverse specimens, including sputum (n=14, 66.7%), blood (n=1, 4.8%), urine (n=3, 14.3%), pleural effusion (n=1, 4.8%), and secretion (n=2, 9.5%). After being isolated and purified, these strains were preserved at -80°C until used for experiments. Meanwhile, the clinical information of these patients was also collected from the Electronic Medical Records. This study was approved by the hospital ethics committee (Approval No: JXEY-2021JX045).

### 2.2 Antibiotic susceptibility and string testing

The minimum inhibitory concentrations (MICs) of 15 antibacterial drugs were tested by broth microdilution method using Biofosun^®^ Gram-negative panels (Biofosun Biotech, Co., Ltd., Shanghai, China). Antibacterial drugs were diluted in sterile cation-adjusted Mueller-Hinton broth. Antibacterial drugs tested were as follows (concentration ranges in parentheses): ertapenem (0.125-128 mg/L), imipenem (0.125-128 mg/L), meropenem (0.125-128 mg/L), ceftriaxone (1-128 mg/L), ceftazidime (1-128 mg/L), cefepime (1-64 mg/L), piperacillin-tazobactam (4/4-256/4 mg/L), cefoperazone/sulbactam (4/2-256/128 mg/L), ceftazidime-avibactam (0.5/4 -4/4 mg/L), polymyxin B (0.125-4 mg/L), tigecycline (0.25-8 mg/L), fosfomycin (1-1024 mg/L), aztreonam (2-128 mg/L), ciprofloxacin (0.5-32 mg/L) and amikacin (1-1024 mg/L).

The breakpoints were set in the light of the Clinical and Laboratory Standards Institute (CLSI, 2016) (except for tigecycline) and the US Food and Drug Administration (for tigecycline) guidelines (Humphries et al., 2021). *Escherichia coli* strain ATCC 25922 was adopted as the quality control (QC) strain. MIC QC values for antimicrobial agents against the QC strain were as follows: ertapenem (<0.125 mg/L), imipenem (0.125-0.25 mg/L), meropenem (<0.125 mg/L), ceftriaxone (<1 mg/L), ceftazidime (<1 mg/L), cefepime (<1 mg/L), piperacillin-tazobactam (<4/4 mg/L), cefoperazone/sulbactam (<4/2 mg/L), ceftazidime-avibactam (<0.5/4 mg/L), polymyxin B (0.5 mg/L), tigecycline (<0.25 mg/L), fosfomycin (1 mg/L), aztreonam (<2 mg/L), ciprofloxacin (<0.5 mg/L), and amikacin (2 mg/L).

To perform the string test, the experimental strain was inoculated onto Columbia agar containing 5% sheep blood and incubated at 35°C for 18-24 h. The inoculation ring gently touched the surface of a single colony and then pulled upward. The string test is considered positive, indicating a hyper viscous phenotype if a viscous string measuring >5 mm in length is obtained by stretching bacterial colonies on an agar plate.

### 2.3 Detection of carbapenemase genes

The common carbapenemase genes (*bla*_KPC_, *bla*_NDM_, *bla*_OXA-48_, *bla*_VIM_, and *bla*_IMP_) from isolates that exhibited non-susceptibility to carbapenems were screened by PCR amplification with primers as described previously and listed in **Table S1** (Poirel et al., 2011). Two microliters of total DNA were subjected to multiplex PCR in a 50-μL reaction mixture. The mix for the detection of *bla*_KPC_, *bla*_NDM_, and *bla*_OXA-48_ genes contains 1× PCR buffer (10 mmol/L Tris–HCl [pH 8.3], 50 mmol/L KCl), 1.5 mmol/L of MgCl2, 0.125 mmol/L of each deoxynucleotide triphosphate, 10 μmol/L of each primer, and 6 ul of Taq Polymerase (Takara). The reaction conditions were as follows: 10 min at 94°C and 36 cycles of amplification consisting of 30 s at 94°C, 40 s at 52°C, and 50 s at 72°C, with 5 min at 72°C for the final extension. Subsequently, the positive PCR products were sequenced and compared with the reported sequences from the NCBI Genbank database via Blast.

### 2.4 Short-read genome sequencing and analysis

Genomic DNA was extracted using a QIAamp DNA mini kit (Qiagen) and subjected to Illumina paired-end sequencing (Illumina Inc., San Diego, CA). Raw reads were trimmed using fastp v0.23.2 (Chen et al., 2018). The trimmed reads were assembled using Unicycler v0.5.0 with default settings (Wick et al., 2017). Gene predictions and functional annotations were performed with Prokka v1.14.5 (Seemann, 2014). Sequence typing, capsule typing and screening for virulence factors and acquired antimicrobial resistance genes were performed using Kleborate v2.0.1 (Lam et al., 2021). MOB-suite v3.0.1 was used to predict plasmid sequences from the hybrid assemblies and identify their replicon types (Robertson and Nash, 2018). The presence of antibiotic resistance (AMR) genes was detected utilizing the CARD and ResFinder databases (Florensa et al., 2022; Alcock et al., 2023). Virulence factors (VF) were predicted with the VFDB database (Liu et al., 2022).

### 2.5 Long-read genome sequencing and analysis

Genomic DNA was extracted using Wizard^®^ Genomic DNA Purification Kit (Promega, Madison, WI) according to the manufacturer’s protocol. The harvested DNA was detected by the agarose gel electrophoresis and quantified by Qubit^®^ 2.0 Fluorometer (Thermo Scientific). Libraries for single-molecule real-time (SMRT) sequencing were constructed with an insert size of 10 kb using the SMRT bell TM Template kit, v1.0 (Pacific Biosciences). DNA samples that passed electrophoresis were fragmented to the desired size for library construction using the Covaris g-TUBE. After DNA damage repair and end repair, DNA ligase was used to connect hairpin adapters to both ends of the DNA fragments. The DNA fragments were purified using AMpure PB magnetic beads, and specific-size fragments were selected using BluePippin. The SMRT Bell library was subjected to concentration screening with AMpure PB magnetic beads, followed by DNA damage repair and another round of purification using AMpure PB magnetic beads. The constructed library was quantified by Qubit, and the insert fragment size was detected using Agilent 2100 before sequencing on the PacBio platform. Raw PacBio long reads were trimmed using filtlong (https://github.com/rrwick/Filtlong) v0.2.1. Hybrid assembly was performed based on trimmed long and short reads using Unicycler v0.5.0 with default settings, resulting in a complete genome assembly (Wick et al., 2017). Plasmid alignment was generated using BRIG v0.95 (Alikhan et al., 2011).

### 2.6 Phylogenetic and transmission analysis

Twelve closely related *K. pneumoniae* genomes were retrieved from the BacWGSTdb server using the core genome multi-locus sequence typing (cgMLST) strategy at a threshold of 150 (Feng et al., 2021). Core genome single nucleotide polymorphisms (cgSNPs) were extracted with Parsnp v1.2, using the most closely related ST15 *K. pneumoniae* complete genome WSD411 (Genbank: ASM988441v1) as a reference (Treangen et al., 2014). Recombination sites were removed using Gubbins v3.3 with default parameters and a starting tree created by Parsnp using a GTR substitution model (Croucher et al., 2015).

Pairwise SNP distances were calculated with SNP-sites v2.5.1 (Page et al., 2016). Furthermore, a cgMLST allele calling was performed with chewBBACA suite using the Prodigal training file for *K. pneumoniae* from cgmlst.org (https://www.cgmlst.org/ncs/schema/2187931/) (Silva et al., 2018). The genomic clustering of all 21 isolates was defined at both cgSNP and cgMLST methods, respectively. For the cgSNP method, a genomic network was constructed with the MSA files with a median-joining network inference method implemented in PopArt software (Leigh and Bryant, 2015).

To estimate the emergence of the most recent common ancestor of our isolates, we further generated a concatenated sequence at the non-redundant SNP loci of our 21 isolates and 12 other closely related genomes using Parsnp and Gubbins as detailed above. The dated phylogeny was inferred using BactDating v1.0.12 under the arc model with 10^8^ iterations to ensure that the Markov chain Monte Carlo was run long enough to converge (Didelot et al., 2018). The phylogenetic tree was visualized with R package ggtree (Yu et al., 2018).

## 3 Results and discussion

### 3.1 Outbreak description of clinical ST15 OXA-232-producing CRKP strains

**Figure 1:**
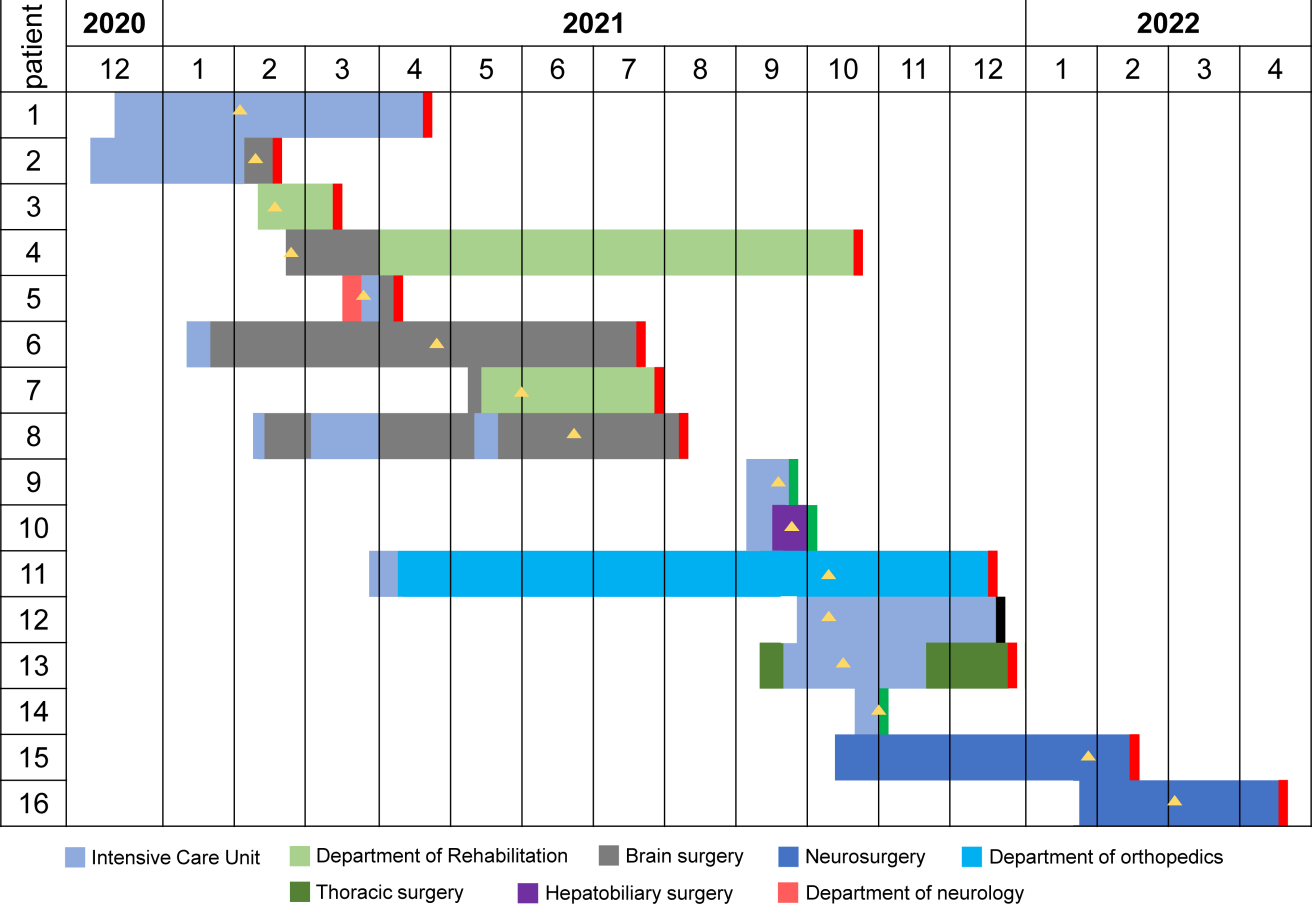
The timeline of ST15 OXA-232-producing CRKP outbreak in south-eastern China. Different wards are indicated in different colors. The black rectangle indicates that the outcome of this patient was death. The red rectangle indicates that the outcome of this patient was cured. The green rectangle indicates that the outcome of this patient was not cured. The yellow triangle indicates the isolation of the CRKP strain.

The Second Affiliated Hospital of Jiaxing University in the northeastern Zhejiang province of China was affected by an outbreak of OXA-232-producing CRKP between February 2021 and March 2022. We recovered 21 OXA-232-producing CRKP isolates from 16 inpatients (**Table S2**). MLST and serotyping analysis revealed that all 21 isolates belonged to ST15 and had capsular KL112 and antigen O2v1 loci. Of the 16 patients, 81.3% (n=13) were male and 18.7% (n=3) were female, with a median age of 62 years (range: 31-87 years). All the patients reported clinical symptoms such as sepsis, pneumonia, or urinary tract infection with severe underlying diseases. These patients were admitted to five different wards (**Figure 1**). These phenomena suggested a major outbreak concern for the hospital infection management department.

A retrospective analysis of the clinical information of the 16 patients indicated that the index patient (Patient 1, P1) was a 70-year-old male admitted to Zhejiang Sian International Hospital on December 15, 2020, for surgery due to a car accident and then was treated in ICU. On the second day post-surgery, the patient was transferred to our hospital for further treatment in ICU. On February 2, 2021, the CRKP was isolated from his sputum sample indicating infection. He was recovered and discharged on April 22, 2021 after effective therapy. Patient 2 (P2) is a 71-year-old male with bilateral pleural effusion and a lung infection after craniocerebral surgery because of injuries from a car accident. He was transferred to the NICU of our hospital On December 8, 2020, for additional care. The CRKP was isolated from his sputum sample on February 9, 2021. After long-term treatment, the patient was transferred to the department of cerebral surgery with an improvement of the disease. The subsequent patients (P3 to P16) were admitted to the ICU and general ward, and the first CRKP was detected on February 18, 2021, in these 14 patients. Four patients (P2, P6, P7 and P11) were transferred from the Second Affiliated Hospital of Zhejiang University School of Medicine in Hangzhou, and two (P15 and P16) were first treated in Shanghai.

### 3.2 Molecular epidemiology of the OXA-232-producing CRKP outbreak

**Figure 2:**
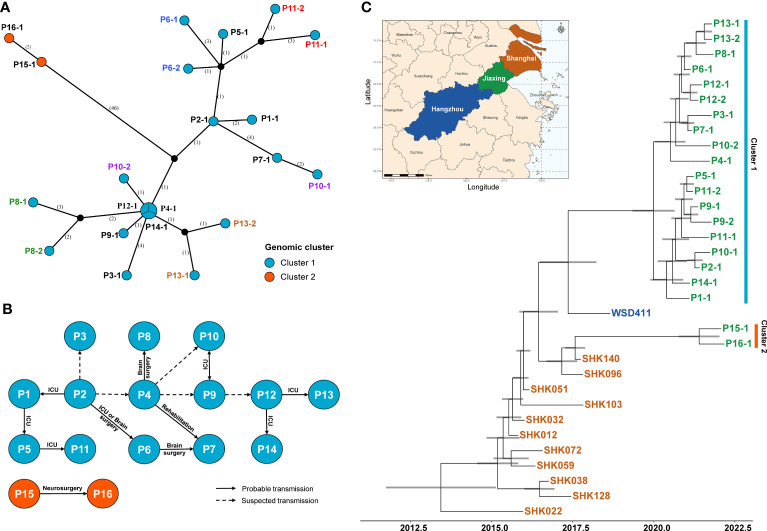
Molecular epidemiology characteristics of the ST15 OXA-232-producing CRKP isolates in Jiaxing. **(A)** Median-joining network constructed with PopART for haplotypes. Circle sizes and colors reflect haplotype abundances and genomic clusters, respectively. Black circles represent unsampled or extinct haplotypes (Leigh and Bryant, 2015). The numbers in parentheses on branches between haplotypes denote their SNP difference. **(B)** Transmission diagram of the outbreak of the isolates in the hospital. The color of each node indicates the genomic cluster. The connecting line between two nodes indicates the epidemiological link, and the arrow indicates the inferred transmission direction. **(C)** Dated phylogenetic tree of the 21 strains in Jiaxing and the 12 closely related strains isolated from Hangzhou and Shanghai. The map on the upper left shows the location of these three cities. The color of the terminal branch node of the phylogenetic tree is consistent with the color on the map, representing the city where the strain was isolated. The time scale is indicated at the bottom.

16 draft and two complete genome sequences were obtained by WGS (**Table S3A**). A haplotype network analysis based on the maximum core genome SNP distance between isolates revealed two genomic clusters (**Figure 2A**). The large cluster (cluster 1) included 19 isolates from 14 patients recovered in 2021, while the small cluster (cluster 2) comprised of two isolates recovered in 2022. The maximum pairwise SNP differences within cluster 1 and cluster 2 were 13 and 2 SNPs, respectively. Although most isolates from the same patient had an SNP distance of no more than 5, we observed 9 SNPs between sputum (P10-1) and secretion (P10-2) isolates from P10. The median-joining network revealed that P10-1 is closest to P2-1, while P10-2 is closer to P3-1, P7-1 and P8-1. We also observed the presence of unsampled cases within the OXA-232-producing CRKP outbreak. A cgMLST profiling also revealed a similar genomic relatedness structure at the alleles (**Figure S1**). The maximum number of alleles within each genomic cluster was no more than 10.

According to the SNP distance between the strains in the haplotype network and the overlap of the environment where the patients were hospitalized, we reconstructed the transmission network of ST 15 CRKP in our hospital (**Figure 2B**). The network contained 11 probable transmission events that met the above two conditions simultaneously and 5 suspected transmission events inferred only based on the SNP distance between strains. The environment where the probable transmission occurred most was the ICU (7 events), followed by brain surgery (3 events), rehabilitation (1 event), and neurosurgery (1 event) department.

P2 was the index case for cluster 1 and then transmitted to P1 in the ICU. Next, the transmission from P1 to P5 and P5 to P11 were both in the ICU. P2 transmitted the infection to P6 in the brain surgery department or ICU. Besides, we hypothesized that the infections of P3 and P4 might also be derived from P2. Then, P4 transmitted the infection to P8 in the brain surgery department. There were two possible transmission routes for P7, from P4 in the rehabilitation department or P6 in the brain surgery department. We also suspected that the infections of both P9 and P10 were related to P4. Both P9 and P10 were admitted on the same date to ICU, there was no certain in whom the transmitter was. The infection of P12 might be derived from P9. Next, P12 transmitted the infections to P13 and P14 in the ICU. The transmission of cluster 2 only occurred in neurosurgery. P15 was the index case and then transmitted to P16.

The increasing amounts of available genomic data have enabled us to investigate the putative source of infection. We identified the other 12 closely related genomes from the BacWGSTdb database at a cgMLST threshold of 150 (**Table S3B**). According to the original research paper, all these isolates were OXA-232-producing CRKP and belonged to ST15. Among these strains, eleven were isolated in a teaching hospital in Shanghai, and the other one was recovered from a teaching hospital in Hangzhou (Li et al., 2019; Han et al., 2022). Jiaxing is situated northeast of Zhejiang, bordering Shanghai to the east and Hangzhou to the west (**Figure 2C**). Additionally, we found that the P15 patient was transferred to Shanghai Hospital for further symptomatic and supportive treatment after emergency surgery in our hospital, and then transferred to our hospital for rehabilitation treatment. Still, then the CRKP was identified from the sputum. This process indicated the possible origin of the clone from a hospital in Shanghai. However, the exact route of the interhospital transmission between Hangzhou and Jiaxing remains unclear.

Moreover, an SNP-based Bayesian phylogenetic analysis was performed to estimate the emergence date of 33 isolates (**Figure 2C**). The most recent common ancestor of our 21 isolates was estimated to emerge in July 2016 (95% confidence interval [CI], December 2015 to February 2017). According to the clustering results, it suggests that the cluster 1 outbreak emerged first in November 2019 (95% CI, March 2019 to June 2020), whereas the cluster 2 outbreak emerged in March 2021 (95% CI, August 2020 to November 2021).

### 3.3 Antibiotic resistance in the isolates

All 21 strains exhibited comparable patterns of drug resistance, encompassing ceftriaxone, ceftazidime, cefepime, piperacillin/tazobactam, cefoperazone/sulbactam, aztreonam, and ciprofloxacin (**Table S4**). Complete resistance was observed against amikacin, whereas they demonstrated complete sensitivity to ceftazidime/avibactam and tigecycline. Moreover, the strains displayed a 95.3% susceptibility rate (20/21) to polymyxin B. The strains also showed different levels of resistance to different carbapenems (ertapenem, imipenem and meropenem). Notably, 85.7% (18/21) of the strains exhibited MICs of ertapenem ≥64 mg/L. Conversely, all strains displayed imipenem MICs of ≤16 mg/L, while meropenem MICs were uniformly ≤32 mg/L.

### 3.4 AMR and VF genes profiles

All CRKP isolates were negative for the string test. Whole-genome screening of the virulence factors from VFDB revealed that these isolates were hypervirulent with the virulence gene *rmpA2* encoding regulators of mucoid phenotype (**Table S5A**). In addition, all 21 isolates had *iucABCD* encoding aerobactin and *ybtAEPQSTUX* encoding yersiniabactin. None of them had the virulence gene *clb* (encoding colibactin). Compared with cluster 1, the VF profiles of cluster 2 had a more VF *mrkH* related to type 3 fimbriae and one less VF *KP1_RS17225* related to the capsule.

Based on the CARD database (**Table S5B**), all 21 CRKP isolates carried the carbapenemase gene *bla*_OXA-232_ and *bla*_CTX-M-15_/*bla*_SHV-28_ encoding an extended-spectrum *β*-lactamase (ESBL). Besides, all 21 isolates harbored *fosA* (mediating fosfomycin resistance), *oqxA*/*oqxB* (mediating quinolone resistance), and *tet(E)* (mediating tetracycline resistance), while 68.4% (13/19) of strains in cluster 1 also harbored the aminoglycoside antibiotic-resistance gene *AAC(6’)-Ib*. Notably, both two isolates in cluster 2 carried four other antibiotics resistance genes: *APH(3’’)-Ib*, *APH(6)-Id* (mediating aminoglycoside antibiotic resistance), *TEM-1* (encoding non-ESBL *β*-lactamases) and *sul2* (mediating sulfonamide antibiotic resistance).

### 3.5 Characteristics of plasmids

**Figure 3:**
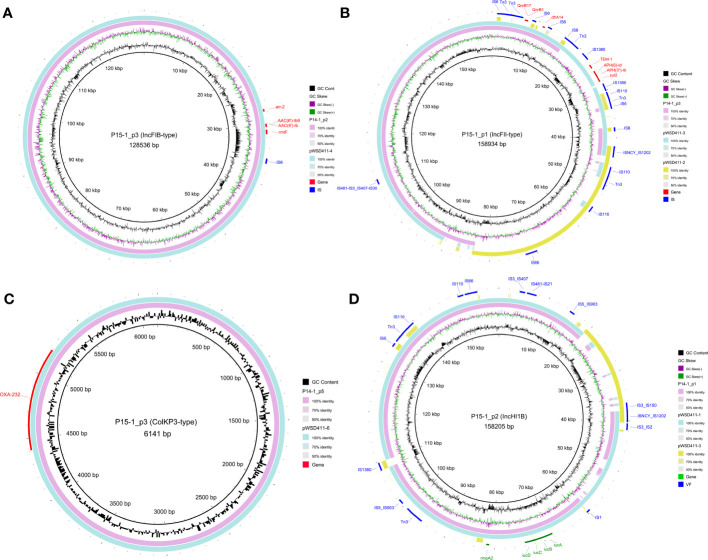
Genomic structure of the plasmids harboring AMR genes or VF genes in OXA-232-ST15 CRKP. **(A)** IncFIB-type plasmids. **(B)** IncFII-type plasmids. **(C)** ColKP3-type plasmids. **(D)** IncHI1B-type plasmids. AMR and VF genes are marked with red and green blocks, respectively. Mobile genetic elements are marked in blue color.

The complete genomes of two representative strains, P14-1 (cluster 1) and P15-1 (cluster 2), were obtained based on a hybrid assembly utilizing long-read and short-read sequencing data. P14-1 and P15-1 harbored the same number of genomic replicons, including one chromosome and nine plasmids (**Table S6**). There was the same content of plasmid clusters between P14-1 and P15-1, including one conjugative plasmid, four mobilizable plasmids, and four non-mobilizable plasmids. There were 12 and 8 AMR genes on the plasmids of P14-1 and P15-1 (**Figure 3**), respectively. The same plasmid-harbored AMR genes were *arr-2*, *AAC(6’)-Ib9*, *AAC(6’)-Ib*, and *rmtF* on the IncFIB-type plasmids (P14-1_p2 and P15-1_p3, **Figure 3A**), *QnrB17*, *QnrB1* and *dfrA14* on the IncFII-type plasmids (P14-1_p3 and P15-1_p1, **Figure 3B**), and *OXA-232* on the ColKP3-type plasmids (P14-1_p5 and P15-1_p5, **Figure 3C**). However, in contrast to the IncFII-type plasmids found in strains WSD411 and P15, P14-1_p3 exhibited the presence of *qnrB* and *dfrA14*. Remarkably, P14-1_p3 was devoid of *blaTEM-1*, *aph(6)-Id*, *aph(3’’)-Ib*, and *sul2*. This discrepancy may be attributed to the insertion mediated by IS1380 transposase. Furthermore, the IncHI1B-type plasmids (P15-1_p2 and P14-1_p1, **Figure 3D**) were the typical virulence plasmids containing determinants of *rmpA2* (hypermucoidy) and *iucABCD* (aerobactin siderophore).

Interestingly, we observed the two plasmid fusions in both large plasmids (P15-1_p1 and P15-1_p2) in P15-1. The IncFII-type P15-1_p1 was a fusion plasmid formed by recombination of the homologous pWSD411-3 and ~42 kbp conjugative IncFIB-type pWSD411-2. The IncHI1B-type P15-1_p2 was also formed by recombination of the homologous pWSD411-1 and ~20 kbp non-mobilizable IncFII-type pWSD411-3. Mobile genetic element analysis revealed that both fusion events occurred via several transposases, including IS1202, IS110, Tn3 and IS3 families. BLAST results showed that it exhibited a high level of sequence identity with each homologous region.

## 4 Discussion

Over the past five years, the incidence of OXA-232-producing CRKP has been increasing in China (Yang et al., 2023). This study describes an outbreak of 21 CRKP strains recovered from patients at a tertiary teaching hospital in Zhejiang Province, China. The outbreak was caused by OXA-232-producing ST15 CRKP. Although OXA-232-producing CRKP is not as prevalent in China as KPC-2-producing *K. pneumoniae*, their multi-drug resistance limits treatment options and potentially threatens global public health.

Recent epidemiological studies on the source of nosocomial infection have shown that hospital outbreaks start from patients themselves (David et al., 2019; Han et al., 2022). Environmental reservoirs such as hospital sinks and toilets are where the infection spreads (Qiao et al., 2020). Besides, healthcare workers are also potential intermediaries (Puig-Asensio et al., 2020). With the advances in WGS-based molecular subtyping methods, we confirmed that these ST15 CRKP strains might originate from two clones. The first clone outbreak was from February 2021 to October 2021, causing 14 patients to be infected. While just two months later, the second clone appeared in January 2022 with two infections. Moreover, we inferred that the earliest occurrences of the outbreaks for at least a year before admission of the index patient. Worryingly, our extrapolated timing of the outbreak preceded the detection of the index case by nearly a year, suggesting that there might have been undetected infections during this period.

This study successfully reconstructed most of the transmission chains of this nosocomial outbreak by combining molecular network and clinical data. We found three main transmission settings of CRKP, with the ICU being the most, followed by the brain surgery, neurosurgery and rehabilitation department. Previous studies show that CRKP is ubiquitousin ICUs (Tzouvelekis et al., 2012). There is evidence that widespread contamination of medical devices, mobile equipment surfaces, and handwashing sinks in the ICU environment plays a role in the nosocomial spread of CRKP (Qiao et al., 2020; Han et al., 2022). Therefore, it is necessary to strengthen the intervention measures for the environment, patients, and medical staff in the ICU to prevent the further spread of CRKP.

In many instances, these transmissions correlated well with the movement of patients through the hospital, but some transmissions could not be predicted by the epidemiological data alone. For example, the isolate of P3 belonged to the transmission chain of cluster 1, but the hospitalization records showed that P3 had been in the rehabilitation department and had not been to both ICUs and brain surgery. Therefore, we are still unable to identify the route by which it was infected. This phenomenon suggests a possible role of the movement of staff, equipment, or further unsampled sources for the nosocomial transmissions, as well as highlighting the need to integrate genetic and epidemiological data to track and reconstruct the CRKP outbreaks fully (Doughty et al., 2023).

Some recent studies have also reported the outbreak and prevalence of the ST15 OXA-232-producing CRKP in hospitals in other cities of Zhejiang province, including Hangzhou, Taizhou, Jinhua, and Wenzhou (Jia et al., 2021; Han et al., 2022; Zhang et al., 2023). Our study provides further evidence of its broad spreading tendency in Zhejiang. Furthermore, through an in-depth database search, we further speculated that the sources of the two outbreaks were the hospitals in Hangzhou and Shanghai, two large cities around Jiaxing. Due to a large number of floating populations, our hospital often treats patients transferred from Hangzhou and Shanghai, so it is urgent to clarify the way of cross-hospital transmission further and strengthen monitoring and management to reduce the spread of carbapenem resistance.

In addition to carrying the carbapenem resistance gene *bla*_OXA232_, all ST15 CRKP strains showed high resistance (>90%) to all beta-lactam antibiotics, including cefuroxime, cefotaxime, cefepime, piperacillin/tazobactam, ceftazidime/avibactam, and aztreonam, except for high sensitivity (100%) to ceftazidime/avibactam and tigecycline. One strain had polymyxin B resistance, but no *MCR-1* gene was detected, indicating additional underlying mechanisms. Previous studies have reported that OXA-232 strains have low-level resistance to carbapenem antibiotics, and higher meropenem sensitivity than imipenem (Poirel et al., 2012; Bakthavatchalam et al., 2016). However, this study found that the MIC of imipenem was 2-16mg/L, lower than that of meropenem (4-32mg/L), which may be related to the presence of membrane protein genes (OmpK35/36/37). The antibiotic susceptibility and resistance genes of ST15-type strains reported in the study also differ from previous reports (Hu et al., 2013; Han et al., 2021; Zhao et al., 2022). Most ST15 OXA-232-producing CRKP strains in previous studies co-produce *CTX-M-15*, *KPC-2* and *NDM-1*, while our strains carry three more AMR genes, including *CTX-M-1*, *SHV-28* and *TEM-1* genes, as well as the aminoglycoside resistance gene 16S rRNA methylase *rmtF*. The composition of these resistance-related genes is consistent with the results of drug susceptibility tests that all strains are resistant to amikacin at a high level. In contrast, previous studies showed that ST15 KPC-2-producing CRKP is 100% sensitive to amikacin., Therefore, there are differences in susceptibility to other antibiotics among ST15 CRKP encoding different carbapenemases, which is caused by the differences in the composition of AMR genes.

Horizontal transmission of mobile elements such as plasmids, phages, integrations, conjugation elements, insertion elements, etc., is a crucial factor in *K. pneumoniae* outbreaks (Zong et al., 2021). Utilizing the long-read sequencing, we recovered the complete plasmid sequences of two clones. The *bla*_OXA-232_ was located on a small ColKP3-type non-conjugative plasmid in both clones. Only ST15 CRKP containing the *bla*_OXA-232_ gene located within the ColKP3-type plasmid has been reported in China. However, this small plasmid does not contain genes sufficient for self-transfer. It could transmit with the help of other conjugative plasmids, such as IncFII-type plasmid P15-1_p1 and P14-1_p3. Therefore, further studies are needed to investigate the transmission mechanism of ColKP3-type plasmids to prevent further transmission to other species.

Both clones also harbored a non-mobilizable virulence plasmid encoding the hypermucoidy gene *rmpA2* and aerobactin siderophore genes *iucABCD*. Aerobactin is associated explicitly with growth in blood and is a stronger predictor of the hypervirulence phenotype. It is worth noting that we observed extensive fragment plasmid fusions in two non-conjugative MDR and virulence plasmids contributed by several transposases. Due to the presence of various mobile elements in both MDR and virulence plasmids, we speculate that these plasmids have a high potential to fuse with other conjugative plasmids and form conjugative MDR and virulence-encoding plasmids, facilitating transmission of virulence plasmid to different types of *K. pneumoniae* strains (Xie et al., 2020; Wang et al., 2022).

One of the limitations of our study was that we only collected clinical strains and did not perform active screening for ICU patients. Therefore, patients who CRKP colonized that might be associated with sinks should have been included. It is strongly advised that samples from nearby provinces and cities be gathered for further research to comprehensively reflect the prevalence of OXA-48-type carbapenemases in China.

Despite these limitations, this study provides valuable evidence that ST15-type CRKP has various types of drug resistance genes, virulence genes widely exist, and the strains in this study have clonal transmission. WGS is a new technique for studying the molecular biological characteristics of pathogenic bacteria. This technology allowed us to better understand the drug resistance and virulence of bacterial strains, trace their origin, as well as to prevent the emergence and spread of clinical multi-drug resistant hypervirulent *K. pneumoniae*.

## 5 Conclusion

In conclusion, we reported an outbreak of ST15 OXA-232-producing CRKP that occurred in our hospital between February 2021 and March 2022. The main settings for nosocomial infection of CRKP were the ICU, followed by the brain surgery, neurosurgery and rehabilitation department. The ST15 CRKP isolates were highly resistant to commonly used antibiotics. They carried multiple *β*-lactamase genes and several multiple aerobactin and yersiniabactin encoding genes, which may play an essential role in the virulence and resistance of ST15 CRKP isolates. Additionally, the phylogenetic tree also suggests that ST15 CRKP may have spread widely in localized areas. Therefore, there is an urgent need to strengthen surveillance and implement strict infection control measures to prevent the widespread of OXA-232-producing CRKP in China.

## Data availability statement

The datasets presented in this study can be found in online repositories. Genome sequences of all strains have been deposited in the NCBI database under BioProject accession numbers PRJNA975316.

## Ethics statement

This study was approved by the Ethical Committee of the Second Affiliated Hospital of Jiaxing University.

## Author contributions

XW and JY conducted the clinical *K. pneumoniae* isolates from outpatients and inpatients, managed the participants, interpreted the data, and drafted and reviewed the manuscript. XCL, MS, CF, YL, and JG analyzed the strains samples, analyzed the strains data, interpreted the data, and reviewed and contributed to the manuscript. XSL and HL conceived the study, analyzed the microbiome data, interpreted the data, and reviewed and contributed to the manuscript. All authors contributed to the article and approved the submitted version.

## Supplementary material

Supplementary Figure 1 Heatmap of all pairwise allele distances based on the cgMLST typing.

Supplementary Table 2 Metadata for 16 inpatients.

Supplementary Table 3 Metadata for the 21 CRKP genomes in this study **(A)** and 12 closely related CRKP genomes from the public database **(B)**.

Supplementary Table 4 Antibiotic susceptibility of 21 CRKP isolates **(A)** and Antimicrobial test ranges, MIC quality control ranges, and MIC breakpoints used for antimicrobial susceptibility testing **(B)**.

Supplementary Table 5 VF **(A)** and AMR **(B)** genes content for each *K. pneumoniae* isolate.

Supplementary Table 6 Plasmids in the complete genomes P14-1 and P15-1.
