# Supplementary figures and images for: Outbreak of OXA-232-producing carbapenem-resistant Klebsiella pneumoniae ST15 in a Chinese teaching hospital: a molecular epidemiological study
Source: Front Cell Infect Microbiol. 2023 Aug 21;13:1229284. doi: 10.3389/fcimb.2023.1229284 (PMC10475586; doi:10.3389/fcimb.2023.1229284)

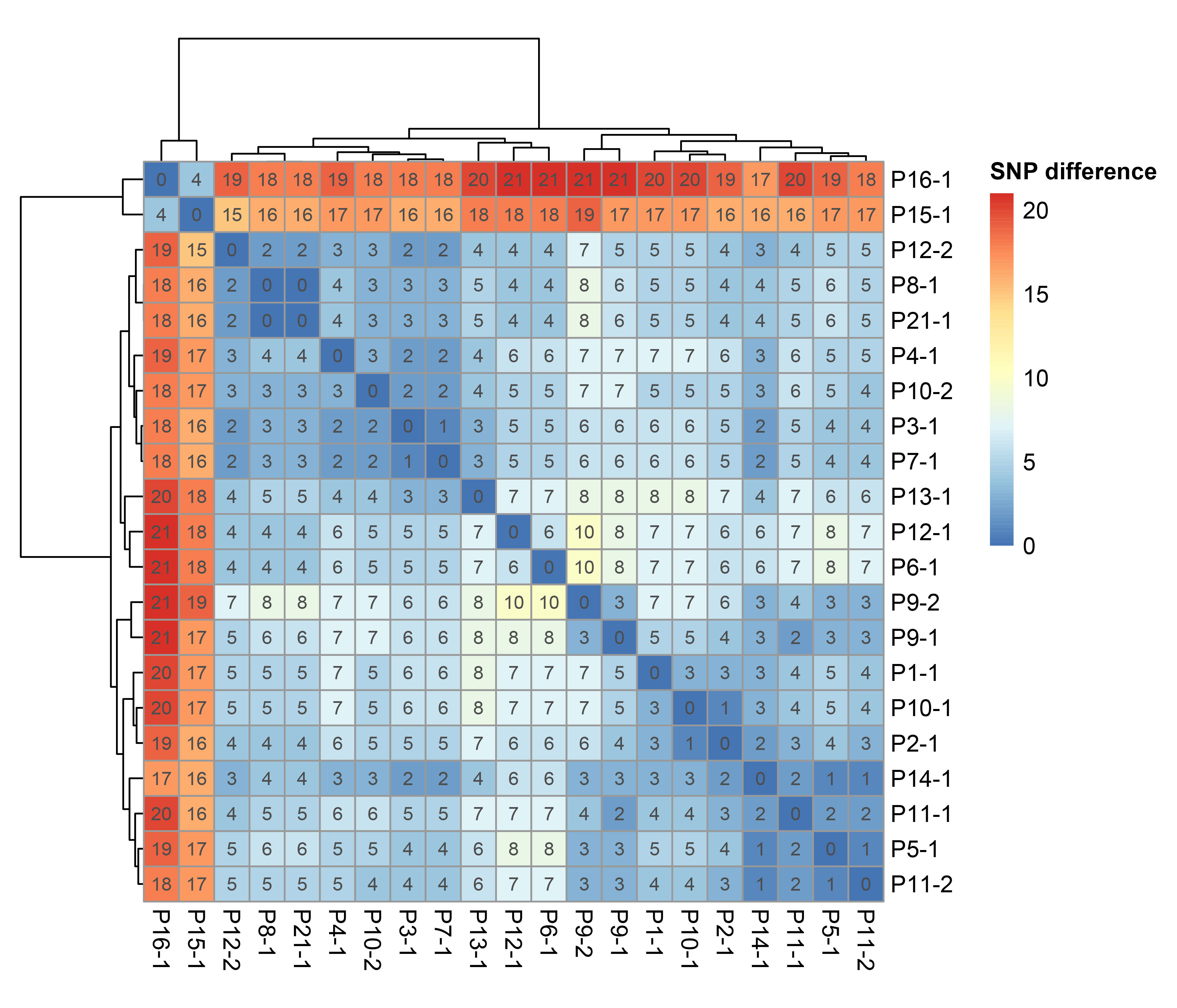

Supplement: Supplementary Figure 1 — Heatmap of all pairwise allele distances based on the cgMLST typing. [file Image_1.jpeg]
