# Supplementary material for: Outbreak of OXA-232-producing carbapenem-resistant Klebsiella pneumoniae ST15 in a Chinese teaching hospital: a molecular epidemiological study
Source: Front Cell Infect Microbiol. 2023 Aug 21;13:1229284. doi: 10.3389/fcimb.2023.1229284 (PMC10475586; doi:10.3389/fcimb.2023.1229284)
Supplement: Supplementary file 2 [file Table_1.docx]

| **Table S1**. **Primers used for PCR detection of carbapenemase genes** | | |  |
| --- | --- | --- | --- |
| **Gene** | **Primer** | **Sequence (5'-3')** | **Product size (bp)** |
| *bla*_OXA-48_ | OXA-48-F | GCGTGGTTAAGGATGAACAC | 438 |
|  | OXA-48-F | CATCAAGTTCAACCCAACCG |  |
| *bla*_NDM_ | NDM-F | GGTTTGGCGATCTGGTTTTC | 621 |
|  | NDM-F | CGGAATGGCTCATCACGATC |  |
| *bla*_KPC_ | KPC-F | CGTCTAGTTCTGCTGTCTTG | 798 |
|  | KPC-R | CTTGTCATCCTTGTTAGGCG |  |
| *bla*_VIM_ | VIM-F | GATGGTGTTTGGTCGCATA | 390 |
|  | VIM-R | CGAATGCGCAGCACCAG |  |
| *bla*_IMP_ | IMP-F | GGAATAGAGTGGCTTAAYTCTC | 232 |
|  | IMP-R | GGTTTAAYAAAACAACCACC |  |
